# Scrotal hematoma following femoral artery puncture

**DOI:** 10.1002/ccr3.1995

**Published:** 2019-01-08

**Authors:** Shun Ishibashi, Kenichi Sakakura, Kei Yamamoto, Tomohisa Okochi, Shin‐ichi Momomura, Hideo Fujita

**Affiliations:** ^1^ Division of Cardiovascular Medicine, Saitama Medical Center Jichi Medical University Saitama Japan; ^2^ Department of Radiology, Saitama Medical Center Jichi Medical University Saitama Japan

**Keywords:** complications, femoral artery puncture, hematoma, inguinal canal

## Abstract

The scrotum hematoma following femoral artery puncture is a rare complication. The bleeding from the puncture site drained through the inguinal canal into the scrotum. The present case may indicate the importance of quick observation of the scrotum, when the puncture of femoral artery was performed.

A 65‐year‐old man with chronic renal failure underwent emergent coronary angiography, because of chest pain. We inserted a 5‐Fr long sheath into right femoral artery under fluoroscopy. As there was no significant coronary stenosis, we removed the femoral sheath and performed manual compression for 20 minutes. His scrotum rapidly grew accompanying severe scrotum pain. Contrast‐enhanced computed tomography (CT) revealed an extravasation of contrast media in the inguinal canal from the right femoral artery (Figure 1, panel A,B). Following additional 30 minutes’ manual compression, the extravasation resolved (Figure 1, panel C,D). We speculated that we might puncture the femoral artery from the medial side to the lateral side (Green line in Figure 1, panel E). Thus, we punctured both the femoral artery and the inguinal canal together. The direct connection between the femoral artery and the inguinal canal occurred.

**Figure 1:**
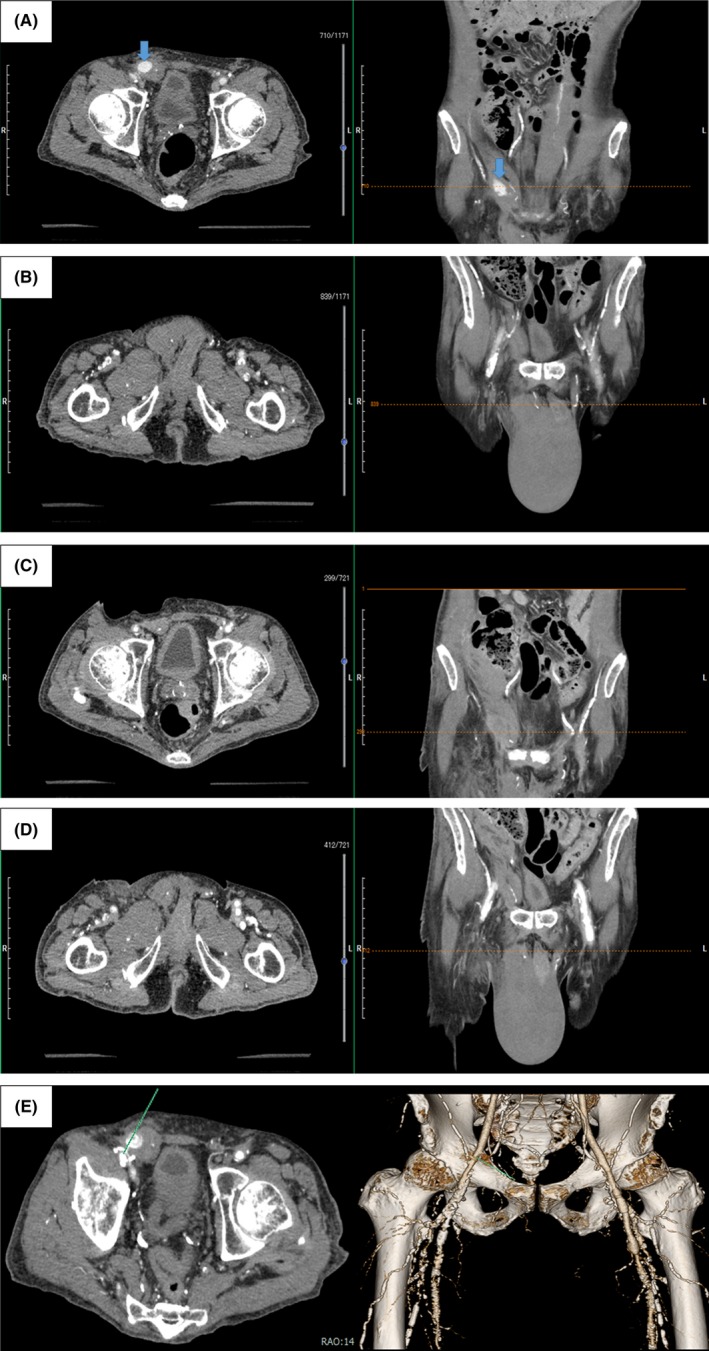
Panel A, Contrast‐enhanced CT after the removal of the femoral sheath and manual compression for 20 min. Blue arrow shows an extravasation of contrast media in the right inguinal canal from the right femoral artery. Panel B, The scrotal hematoma located center of pubis. Panel C, Contrast‐enhanced CT after additional 30 min manual compression. An extravasation of contrast media resolved. Panel D, The scrotal hematoma was not enlarged after additional 30 min manual compression. Panel E, Contrast‐enhanced CT after the removal of the femoral sheath and manual compression for 20 min. Green line is a speculated puncture line. Green line penetrated both the inguinal canal and the femoral artery

The scrotal hematoma can happen following testicular torsion, adrenal hemorrhage, or birth trauma.^1^ Moreover, idiopathic scrotal hematoma was also reported.^1^, ^2^ To the best of our knowledge, our case was the first report regarding scrotal hematoma following puncture of the femoral artery. The present case may indicate the importance of quick observation of the scrotum, when the puncture of femoral artery was performed.

## CONFLICT OF INTEREST

None declared.

## AUTHOR CONTRIBUTIONS

SI: provided medical care, gathered materials for submission, and drafted the initial version of manuscript. KS: supervised SI's work and drafted the manuscript. KY: provided medical care and reviewed the literature. TO: interpreted the CT images and the pathophysiology of the complication. SM: supervised the study. HF: supervised the study. All authors read and approved the final version of the manuscript.
